# Unveiling Lepromatous Leprosy in a Non-endemic Region: A Case of Delayed Diagnosis and Effective ROM (Rifampin, Ofloxacin, and Minocycline) Therapy

**DOI:** 10.7759/cureus.102269

**Published:** 2026-01-25

**Authors:** Maclaine McCarter, Kiley Hassevoort, Jordan Lane, Jonathan L Cleaver

**Affiliations:** 1 Dermatology, A.T. Still University - Kirksville College of Osteopathic Medicine, Kirksville, USA; 2 Medical School, A.T. Still University - Kirksville College of Osteopathic Medicine, Kirksville, USA; 3 Dermatology, Northeast Regional Medical Center, Kirksville, USA; 4 Dermatology, Unity Health - White County Medical Center, Searcy, USA; 5 Dermatology, Cleaver Dermatology, Kirksville, USA; 6 Dermatology, Northeast Regional Medical Center/Still Opti, Kirksville, USA

**Keywords:** diagnostic challenge, hansen’s disease, lepromatous leprosy, leprosy, mycobacterium leprae, rom therapy, rural united states

## Abstract

Leprosy, or Hansen’s disease, is a chronic granulomatous infection caused primarily by *Mycobacterium leprae*. Although its global incidence has declined since the introduction of multidrug therapy (MDT), the disease remains endemic in regions such as India, Brazil, and Indonesia. Additionally, sporadic cases continue to occur in non-endemic areas, including rural areas of the United States, due to migration and travel. We report a 62-year-old male who presented with erythematous, edematous plaques on the arms and trunk, along with progressive hand numbness. He also had a history of frequent travel to Mexico, with the most recent trip nearly a decade prior. After initial misdiagnosis and treatment failure, repeat biopsies revealed numerous Fite-positive acid-fast bacilli. After polymerase chain reaction (PCR) confirmed *M. leprae*, the diagnosis of lepromatous leprosy was established. The patient was treated with a multidrug regimen consisting of rifampin, ofloxacin, and minocycline (ROM therapy) for 24 months, achieving significant clinical improvement within eight weeks without any significant adverse effects.

This case demonstrates leprosy’s long incubation period and its ability to mimic other chronic dermatoses, contributing to diagnostic delays. Histopathology combined with PCR was essential for diagnostic confirmation. ROM therapy demonstrated a favorable treatment outcome, presenting a well-tolerated alternative to the World Health Organization’s standard MDT regimen with rifampin, dapsone, and clofazimine. Clinicians should maintain a high index of suspicion for leprosy in patients with chronic, non-healing skin lesions, particularly when there is a history of travel to endemic regions, even many years prior. Further research comparing ROM and MDT regimens is warranted.

## Introduction

Leprosy, or Hansen’s disease, is a chronic granulomatous infection most commonly caused by *Mycobacterium leprae*. The disease primarily affects the skin and nerves but can also result in widespread, systemic involvement. Leprosy presents as a spectrum of clinical forms, with “multibacillary” lepromatous leprosy (LL) and “paucibacillary” tuberculoid leprosy (TT) representing the two poles of the spectrum [1]. TT is often the result of leprosy disease in patients with effective immunity. This subtype presents with localized and asymmetric hypopigmented papules and plaques, accompanied by decreased sensation in the affected skin [2]. LL represents the multibacillary subtype, characterized by a defective cell-mediated immune response to *M. leprae,* resulting in a high bacillary load. LL typically presents with widespread, symmetric red-brown cutaneous lesions and nerve involvement. Other clinical features of LL include symmetric cushion-like lesions of the face, resulting in “leonine facies,” along with loss of eyelashes and eyebrows. Involvement of the mucous membranes can also be a common finding in LL [2].

Since the introduction of multidrug therapy (MDT) in 1982, the prevalence of leprosy has declined significantly [3]. However, leprosy remains endemic in regions such as India, Brazil, and Indonesia, which together account for roughly 88% of all new global cases [4,5]. In addition, several cases continue to be reported in non-endemic areas due to migration and travel.

Leprosy is known for its insidious onset, which can delay diagnosis, especially in areas of low incidence. In endemic regions, the average incubation period is four years, but it can last over a decade [2]. Diagnosis relies heavily on clinical suspicion and is confirmed by histopathological and bacteriological examination. An intradermal lepromin skin test may aid in diagnosis, although it is often falsely negative in patients with LL [2]. Due to its ability to present in several different ways, the differential diagnosis can be broad but mainly includes other granulomatous, infectious, and neoplastic conditions, such as sarcoidosis, cutaneous leishmaniasis, and cutaneous lymphomas [6].

Early recognition and treatment are essential in the workup of leprosy and in preventing its spread. The standard MDT treatment for LL includes the use of rifampin, dapsone, and clofazimine administered for at least 12 months [3]. Alternative treatment regimens, including fluoroquinolones and minocycline, have gained popularity due to similar treatment outcomes and fewer adverse effects. One such alternative regimen, consisting of rifampin, ofloxacin, and minocycline (ROM therapy), has demonstrated similar efficacy to MDT with milder adverse effects [7]. Despite advances in the treatment of LL, the disease continues to be a significant cause of morbidity, highlighting the need for clinical vigilance and public health efforts. In this report, we present a rare case of lepromatous leprosy occurring in rural Missouri with successful treatment using ROM therapy.

## Case presentation

**Figure 1:**
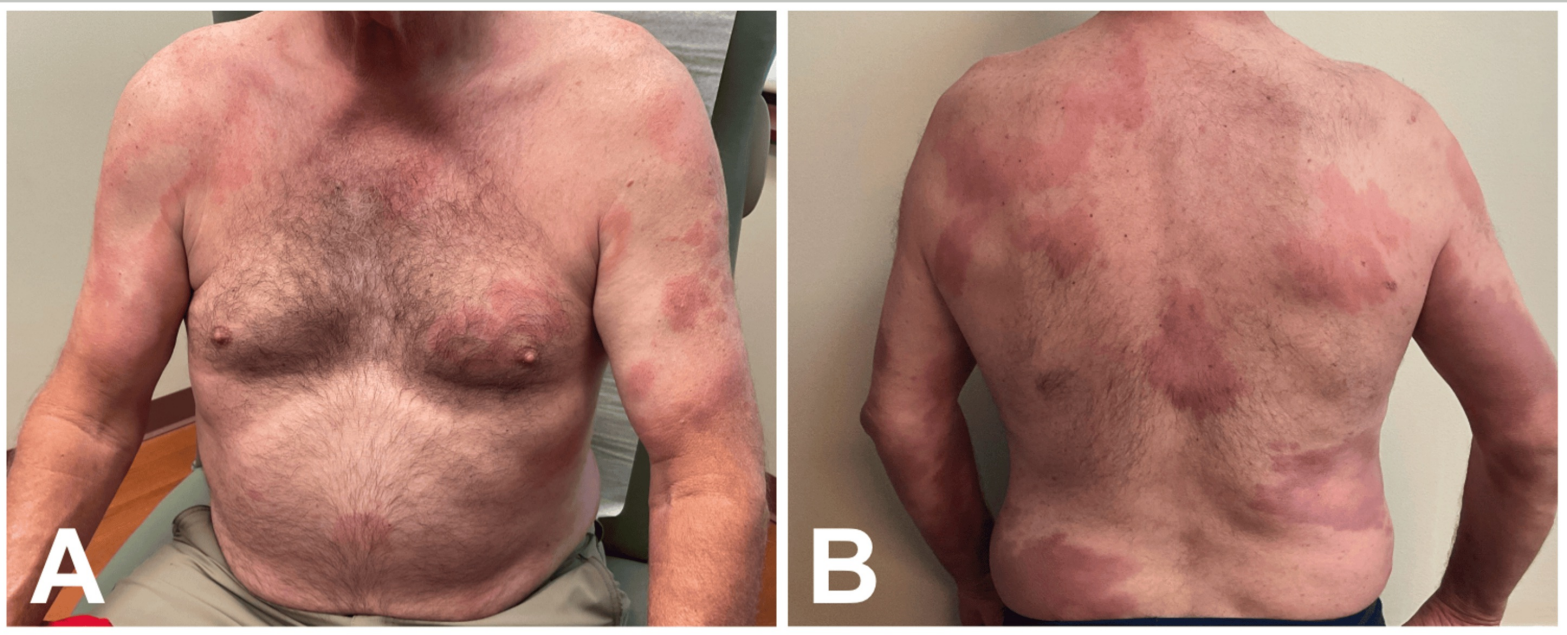
(A) Anterior chest and (B) posterior trunk prior to initiation of ROM therapy. Multiple erythematous, edematous plaques with central clearing are present, consistent with active lepromatous leprosy (LL). Images courtesy of the treating dermatologist. Patient consent was obtained for publication. Non-relevant skin markings (nevi) were obscured to prevent patient identification; leprosy lesions remain unaltered.

A 62-year-old male patient presented to a rural dermatology clinic in Missouri, a region where leprosy cases are extremely uncommon, with erythematous and edematous plaques located on his arms and trunk (Figure 1A, B). The lesions first appeared 3 months prior, and some of the plaques had central clearing. History revealed frequent international travel to Mexico, often twice a year, with his most recent trip occurring nine years before presentation. In Mexico, he spent most of his time with local residents, attending barbecues, visiting bars, and eating local food.

The patient’s only systemic symptom was fatigue, and he also noticed newly developed numbness in both hands that began a few months after the onset of his lesions. At that time, the patient reported no changes to his medications, except for the recent cessation of metformin. The differential diagnosis for his rash included mycosis fungoides, erythema annulare centrifugum, erythema chronica migrans, and urticarial drug eruption.

Two 4-mm punch biopsies were taken. Histopathology favored erythema annulare centrifugum, but he had not been taking any known triggering drugs, except for long-term aspirin use. The patient was instructed to apply 0.1% triamcinolone acetonide cream twice daily and take daily oral antihistamines as needed for itch relief.

One month later, the patient returned with no improvement and the development of new lesions. Two new biopsies were taken from the new lesions. This time, histopathological evaluation showed aggregations of foamy and epithelioid histiocytes containing numerous Fite-positive acid-fast bacilli at all levels of the dermis. Polymerase chain reaction (PCR) was also performed on one of the tissue samples, which confirmed the presence of *M. leprae*. Subsequently, the diagnosis of leprosy, lepromatous type, was confirmed.

Upon receiving these results, the patient was started on combination ROM therapy, as recommended by the National Hansen's Disease Research Laboratory. The treatment regimen followed standard ROM therapy dosing: 600 mg of rifampin, 400 mg of ofloxacin, and 100 mg of minocycline, all administered once monthly for 24 months.

**Figure 2:**
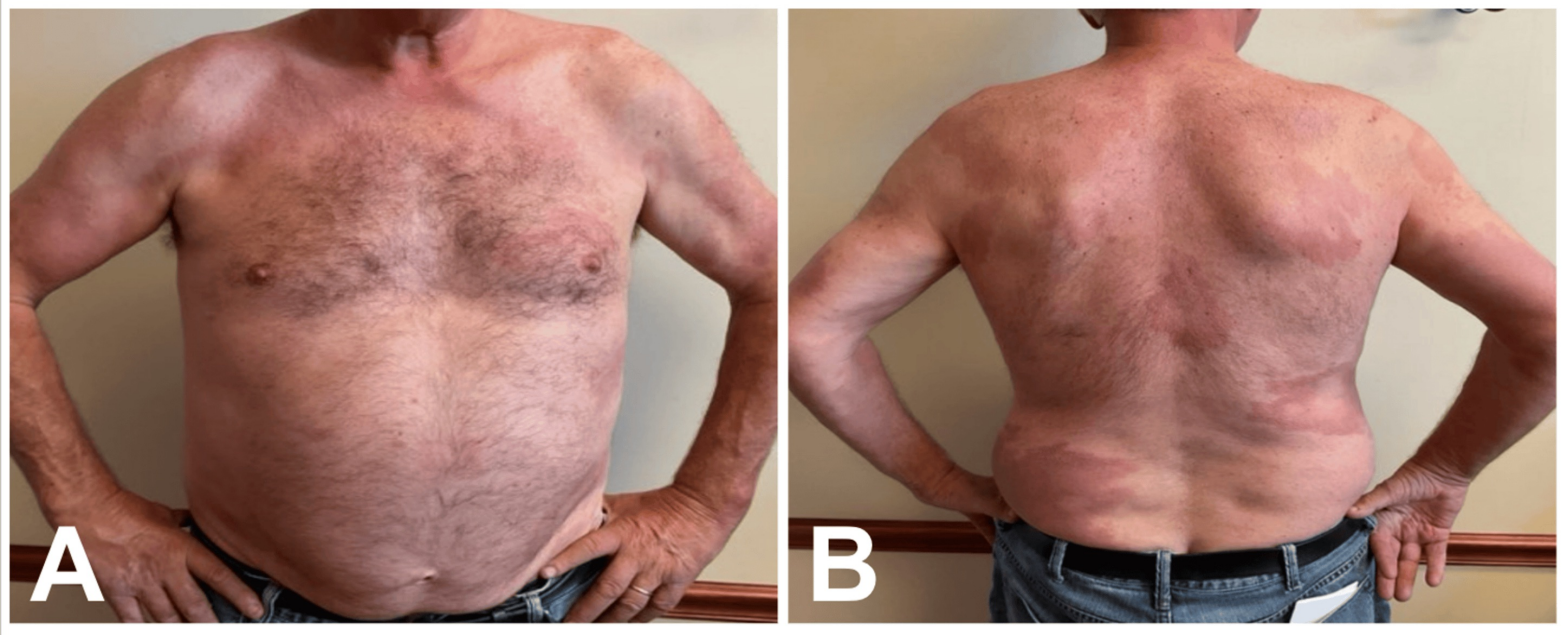
(A) Anterior chest and (B) posterior trunk approximately 6 weeks after initiation of ROM therapy. Partial resolution of erythema and reduction in plaque thickness are observed, demonstrating early clinical improvement. Images courtesy of the treating dermatologist. Patient consent was obtained for publication. Non-relevant skin markings (nevi) were obscured to prevent patient identification; leprosy lesions remain unaltered.

**Figure 3:**
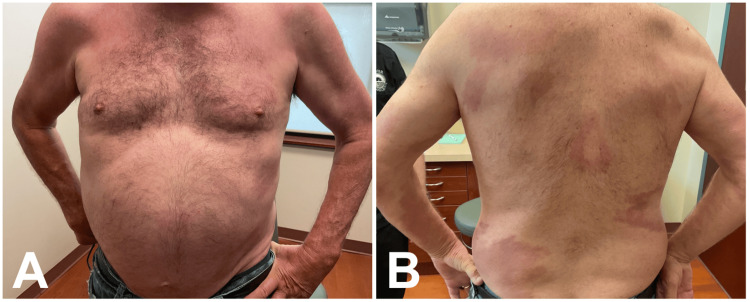
(A) Anterior chest and (B) posterior trunk approximately 8 months after initiation of ROM therapy. Marked improvement with near-complete resolution of plaques and minimal residual erythema is evident, consistent with sustained treatment response. Images courtesy of the treating dermatologist. Patient consent was obtained for publication. Non-relevant skin markings (moles) were obscured to prevent patient identification; leprosy lesions remain unaltered.

After just four weeks of treatment, the patient reported notable improvement in his hand numbness. After six weeks of treatment, the patient noticed improvement in his skin lesions (Figure 2A, B), and further improvement was reported after eight months of treatment (Figure 3A, B). Over the course of treatment, he reported no significant side effects.

## Discussion

Leprosy is a bacterial infection caused by *M. leprae* that is well known for its long incubation period and variable clinical presentation, often leading to delayed diagnosis. This patient’s lesions did not begin to develop until nearly a decade after his last trip to Mexico, which is consistent with reported incubation periods ranging from years to decades [2]. In addition to the long incubation period, leprosy can clinically mimic other chronic dermatologic diseases, as evident in this case. This patient’s erythematous plaques provided minimal specificity for leprosy, leading to a broad range of initial differential diagnoses. Both the prolonged incubation period and the lack of specific findings for leprosy contributed to significant difficulty in establishing a timely diagnosis.

Leprosy requires a high index of suspicion based on history, clinical features, and risk factors, such as international travel, as seen in this patient. The progression from an initial misdiagnosis to a confirmed case of LL emphasizes the importance of maintaining a broad differential diagnosis, especially when patients are refractory to initial therapy. Although leprosy is not endemic to the United States and is exceptionally rare in rural regions such as Missouri, clinicians should maintain a high level of suspicion in patients with chronic, non-healing plaques, particularly when there is a history of travel to or residence in endemic areas.

Histopathological examination showed abundant Fite-positive acid-fast bacilli within dermal histiocytes, which aligns with the characteristic histopathology of LL [8]. Additional PCR testing of the tissue further reinforced these findings, highlighting the importance of using multiple diagnostic tests to confirm leprosy.

Standard treatment for LL consists of MDT, which combines rifampin, dapsone, and clofazimine. However, alternative regimens, such as ROM therapy used in this patient, have previously demonstrated comparable efficacy with fewer adverse effects [7]. The combination of rifampin, ofloxacin, and minocycline provided this patient with relief of both neuropathic and dermatologic manifestations within just 2 months of treatment initiation. In addition, the patient reported no adverse effects. This case adds to the growing body of literature supporting ROM therapy as a safe and effective treatment option.

This case underscores several important considerations in the evaluation of leprosy. First, it illustrates how leprosy can remain dormant and manifest several years after exposure. Second, it highlights the diagnostic challenge posed by early clinical manifestations that can mimic more common dermatoses. Third, it reinforces the importance of further investigation and specialized testing in patients whose clinical course does not follow the expected trajectory. Finally, it demonstrates how ROM therapy can serve as an effective treatment option alongside traditional multidrug regimens. Awareness of these factors is essential to avoid missed diagnoses and to ensure timely and effective treatment.

## Conclusions

Leprosy, a granulomatous disease caused by *Mycobacterium leprae* infection, remains important to consider when forming a differential diagnosis in patients with chronic plaques or nodules. A thorough patient history, including remote travel, is essential in identifying risk factors for rare skin diseases such as leprosy. This case highlights how the prolonged incubation period and nonspecific findings of leprosy can contribute to delays in diagnosis. Clinicians practicing in both endemic and non-endemic regions should maintain vigilance when evaluating chronic skin lesions that are refractory to treatment. While the traditional MDT regimen remains the standard of care for LL, this case demonstrates that ROM combination therapy can be an effective treatment alternative. Continuous awareness and consideration of leprosy are crucial to ensure prompt diagnosis and management.
